# Health Investment Management and Healthcare Quality in the Public System: A Gender Perspective

**DOI:** 10.3390/ijerph18052304

**Published:** 2021-02-26

**Authors:** María del Carmen Valls Martínez, Alicia Ramírez-Orellana, Mayra Soledad Grasso

**Affiliations:** Department of Economics and Business, University of Almería, 04120 Almeria, Spain; aramirez@ual.es (A.R.-O.); mayragrasso21@gmail.com (M.S.G.)

**Keywords:** national health service, healthcare quality, patient satisfaction, health policy, gender perspective, partial least squares structural equation modelling (PLS-SEM)

## Abstract

The aim of this empirical research was to provide useful information for health system managers on the costs and investments involved in improving the quality of the National Health Service (NHS) based on patient assessments and from a gender perspective, i.e., without assuming that the perceived experience is identical for men and women. A cross-sectional study of 31 variables was applied using partial least squares structural equation modeling (PLS-SEM) as a research tool. The data were obtained from the Spanish Ministry of Health, Consumption, and Social Welfare for the entire Spanish territory between 2005 and 2018. The influence of expenditure, resource allocation, and mortality was hypothesized with regard to patient satisfaction according to disconfirmation theory. Patient satisfaction reflects clinical effectiveness, and therefore is a measure of health system quality. The results show that women are more sensitive to public investment in health than men, i.e., an increase in the level of spending and resources increases satisfaction more in women. In both sexes, the level of expenditure has a direct influence on patient satisfaction, and therefore on the quality of the healthcare system. It is important to increase spending on primary care, especially on specialized medical care and diagnostic equipment. However, reducing the use of drugs in favor of alternative treatments or therapies is considered to be positive. Likewise, spending has an impact on available resources, and these, in turn, have a positive influence on the level of use and a negative impact on mortality. Resources, especially healthcare staff, nuclear magnetic resonance equipment, and the number of posts in day hospitals, increase patients’ positive perception of the NHS.

## 1. Introduction

Health is an essential issue in all countries and a complex concept due to its multidimensional nature. Regardless of the socioeconomic level, in many countries, the National Health Service (NHS) guarantees access to health services, thus ensuring equal treatment for all citizens. This contributes to the construction of a prosperous society. Indeed, when the life expectancy of citizens is longer and their health improves, the productive system becomes more efficient, resulting in a stronger economy. This, in turn, will allow an increase in healthcare spending, which will lead to improvements in the health and quality of life of citizens [1]. Consequently, administrations and authorities contribute to achieving continuous improvement in the service provided [2]. According to the World Health Organization (WHO), periodic review of the NHS contributes to improving its performance, which is a fundamental aspect of any society [3]. In this sense, and given that the aim is to enhance citizens’ quality of life, patient evaluation is a key factor in the analysis of healthcare system quality [4].

World economies at all times work to be efficient. Especially in Spain, budgetary restrictions are becoming more frequent, therefore, making efficient use of state resources is one of the most salient points to be addressed [3]. The economic recession of 2008 generated budget cuts in all social services, including the health system. In 2012, the state reduced the health budget by approximately 14% [5]. That, along with the increasing demand for health service in quantity and quality, is why it is even more critical to manage the available resources [6]. The industry, in general, is increasingly customer-oriented. It is important to understand that learning about customer satisfaction is a key to business success [7,8,9]. In the particular case of healthcare, special attention is paid to the patient’s experience throughout the process (admission, investigation, examination, treatment, discharge, and monitoring) [10,11]. It is essential to emphasize not only needs but also patients’ expectations [12,13]. Many times, citizens do not pay much attention to certain public services (e.g., adequate road lighting, cleanliness of public sidewalks, etc.). However, this does not usually happen with the health system, since quality of life is at stake, and even life itself. Even customers in that type of service are more intolerant of the quality service [14].

The National Health System is an international benchmark in terms of universality, accessibility, and effectiveness [15]. According to Numbeo [16], Spain climbed up on the Health Care Index by Country 2019, rising from seventh to sixth place worldwide, while maintaining third place at the European level. The Health Care Index estimates the overall quality of the health care system, health care professionals, equipment, staff, doctors, and cost, among other factors.

In Spain, there is a public health system. The state guarantees access to health services regardless of the socioeconomic level of people who inhabit the country. This allows equal treatment for all. It contributes to the construction of a prosperous society, where citizens’ life expectancy is raised, and at the same time improves economic efficiency [1].

System feedback, focusing on patients, provides information for decision-making and health system improvement [17,18]. Often, the management of health centers focuses on professionals (doctors, nurses, and staff) and not on patients [19]. Nevertheless, considering information on users’ evaluations is a competitive advantage [8]. Incorporating patients’ opinions into management to obtain the modus operandi that improves service provision in the medium or long term [12,20,21] makes the healthcare system more responsive to patient needs. That is, considering patients’ complaints allows for system improvement [22].

Therefore, to continue offering quality service (effective and efficient), managers need to allocate costs adequately (investing in hospital beds is not the same as investing in day hospital posts, specialist physicians, or family physicians, etc.), which requires an optimal application of management strategies in line with proposed objectives [9,23]. The public budget allocated to healthcare puts a limit on the expenses it incurs to continue providing quality service. However, previous studies indicated that quality and efficiency are not mutually exclusive. It is possible to reallocate resources without compromising satisfaction [24] and the quality of healthcare services [25]. In conclusion, the main challenge facing the health system is to provide social welfare with limited and often scarce resources, especially in times of budget adjustments resulting from economic crises. 

Koos [26] and Donabedian [27], in 1954 and 1966, respectively, were pioneers in considering patient feedback as a measure of healthcare outcomes. Later, in 1982, Gronroos first suggested the concept of perceived service quality [28], in terms of patient satisfaction being identified with clinical effectiveness. In fact, this was adopted by the European Foundation Quality Management (EFQM) and the International Organization for Standardization (ISO) [20]. Collecting and analyzing health system data provides information on the aspects that need to be strengthened in order to increase satisfaction, and thus the quality of the health system. This information is necessary in order to adopt the appropriate measures and establish the correct strategies [18,20,21,29,30,31,32]. With proper quality management, the system can be more efficient; that is, it can have more quality at the lowest possible cost [33].

Assessing the satisfaction of a service such as healthcare is complex because it has certain characteristics that make it special. Namely, it is a necessary service that cannot be avoided, and patients have to give up their privacy to the medical staff [34]. Previous studies have shown that patient outcomes are improved, and therefore patients are more satisfied, when they are informed about their options and actively participate in the selection of treatments to be applied in agreement with physicians [6,12]. The literature states that it is an overly complex service [14,35] in which, in addition to other factors, wrong practice poses significant risk to patient health [36]. Evaluating the system’s quality through patient satisfaction will highlight existing deficiencies, and, in this way, they can be corrected to reduce future risks [13].

The concept of patient satisfaction is complex [11,37] and can be understood as the difference between the patient’s expectations and the actual outcome of the healthcare service [4,29,38,39,40]. In short, patient satisfaction is considered a crucial indicator to measure the quality of the service provided [6,30,31,41]. Patient satisfaction can only be improved when the organization knows its needs and expectations, for which it is essential to apply complete quality control and management.

Most of the patient satisfaction studies developed so far were aimed at providing information to healthcare staff (mainly doctors and nurses) on their behavior and relationships with patients (communication, privacy, treatment by and professionalism of the medical staff, received information, etc.) [3,6,17,42]. However, the aim of this study was to provide useful information for health system managers on the costs and investments involved in improving NHS quality based on the assessment of users (patients) and from a gender perspective, i.e., without assuming that the perceived experience is identical for men and women.

The remainder of this paper is organized as follows. The next section contains a review of the literature and the hypotheses established. The second section shows the research methodology. The third section presents the results of the research. Finally, the last section discusses the results achieved and presents the conclusions.

### Literature Background and Hypotheses

Customer (or patient, in the healthcare system) satisfaction is a complex concept that has been the subject of numerous debates in the fields of marketing, psychology, and even philosophy. However, it is not the purpose of this paper to analyze the different conceptions of the term [43].

Satisfaction can be conceived as the result of cognitive information processing, i.e., a comparison of expectations with the perceived performance of the service. This is what in psychology is called disconfirmation theory, a paradigm that has dominated the consumer satisfaction literature since its origins in the early 1970s [44]. Confirmation of expectations occurs when the outcome of the service matches what was initially expected. On the other hand, negative disconfirmation occurs when the result obtained is less than expected, giving rise to dissatisfaction, while positive disconfirmation occurs when the result exceeds initial expectations, causing a feeling of satisfaction [45].

There are two methods for applying this theory [46]. The first, called the inferred method, involves computing the difference between the expectation of performance and the perception of the result obtained. The second, known as the direct method, involves direct measurement of the discrepancy between expectation and perception, with the respondent directly determining the magnitude of the difference. Generally, as in this study, the direct method is used. The EFQM model considers that patient satisfaction represents 20% of the total value of healthcare system quality [38]. Therefore, patients’ opinions represent a main driver of NHS quality.

Satisfaction is a highly subjective concept, thus there is no standardized method to measure patient satisfaction [23,43] and its measurement presents difficulties [37,43]. The importance of patient satisfaction research is that high satisfaction is associated with better clinical outcomes [47] and thus system quality. Some authors state that it may be a “cause–effect” relationship because satisfied patients may be more adherent to treatment and thus achieve better clinical outcomes [48]. For example, Chia confirmed that patient participation in the process of diagnosis and the degree of patient involvement in healthcare decision-making are associated with patient satisfaction [49].

Previous literature indicated that patient satisfaction is related to the development of specific personal skills involving respectful treatment [3], the physician’s behaviors, generating a relationship in the context of education, empathy, courtesy, and respect [10], and the motivation and competence of health professionals [2]. However, such variables are not the subject of our study since they are not related to health spending and investment policies.

We found no evidence of a solid previous literature on studies of patient satisfaction with the NHS differentiated by sex. Nor is there any theory on which to base the different behavior of men and women in relation to the variables analyzed in this work on an individualized basis. Therefore, in this sense, the analysis developed is exploratory and it is only possible to establish a general hypothesis to test a different assessment in men and women. In the future, and based on the results obtained, specific behavioral hypotheses can be established for each variable analyzed.

The relationship between expenditure and satisfaction is positive and significant [3,20,50]. Law 14/1986 granted to the autonomous communities competence in terms of healthcare, and according to the health account system in Spain, health expenditure represented 9.1% of gross domestic product (GDP) in 2016. If we distinguish by autonomous communities, we can see that communities with high per capita health expenditure (Basque Country, Principado de Asturias, and Extremadura) have high satisfaction. On the other hand, communities with lower per capita health expenditure (Andalusia, Madrid, and the Balearic Islands) have lower satisfaction [51]. The expenditure budget applies to direct consumption in a certain period and investments (e.g., in medical facilities and equipment). Therefore, it is reasonable that higher spending will result in greater available resources. Based on the above literature and arguments, we can state the following two hypotheses:

**Hypothesis** **1** **(H1).** *Expenditures positively influence patient satisfaction.*

**Hypothesis** **2** **(H2).** *Expenditures positively influence resource volume.*

Resource allocation is intimately linked with efficiency [33] and is therefore an important variable to analyze, mainly due to its characteristic of being limited. If we obtain information about resource allocation and the measures to take for optimal use, the healthcare system’s overall performance can improve [52]. The previous literature agrees that, for high patient satisfaction, it is necessary to have a healthcare system with adequate infrastructure and medical equipment [9,25,40]; qualified and expert doctors, nurses, and staff; diagnostic facilities and ambulance services [29]; and laboratory services [23]. Kamra et al. [33] revealed the relationship between patient satisfaction and aspects like infrastructure, interpersonal relations, and environmental and functional factors. Handayani’s research was based on the relationship between patient satisfaction and six dimensions: tangibles, responsiveness, reliability, assurance, empathy, and professionals [34]. Some studies confirm the logical assertion that the volume of available resources directly affects the level of use of health services. It stands to reason that, if users have more resources at their disposal, they will use the system more frequently [53]. Therefore, we propose the next hypotheses:

**Hypothesis** **3** **(H3).** *Resource volume positively influences patient satisfaction.*

**Hypothesis** **4** **(H4).** *Resource volume positively influences the extent of use.*

Quality of life is related to physical and psychological aspects, and therefore the risk of mortality [54]. For its part, the quality of the health system directly affects the mortality and quality of life of citizens [25,55]. In this sense, for example, the availability of resources, such as physicians and nurses, will reduce mortality [56,57,58]. Some studies verified that patients with a high risk of mortality are more satisfied than those with a lower risk of mortality [59,60]. The latter could be due to patients’ necessary dependence on the health system [60]. Other research found a weak relationship between health condition and satisfaction [48,61]. However, in general, studies have found a negative relationship between mortality and patient satisfaction [62,63,64]. Consequently, we establish the following hypotheses:

**Hypothesis** **5** **(H5).** *Resource volume negatively influences mortality.*

**Hypothesis** **6** **(H6).** *Mortality level negatively influences patient satisfaction.*

With regard to GDP and distinguishing between autonomous communities, a 2016 study revealed that, in communities with a high GDP per capita, citizens have a better perception of satisfaction [20]. The macroeconomic variable GDP per capita is a good indicator of satisfaction, being a positive relationship [65]. It is more possible for the most productive countries to have a population satisfied with healthcare [50]. High public expenditure on more sophisticated sanitary facilities or the latest equipment may generate greater user satisfaction [65]. Hence, we propose the following hypothesis:

**Hypothesis** **7** **(H7).** *GDP volume positively influences patient satisfaction.*

When a variable interferes between two related variables, a mediating relationship is established. Specifically, this implies that a change in the independent variable results in a change in the mediating variable, which, in turn, changes the dependent variable. Analyzing the intensity of the relationships of the mediating variable with the other two variables makes it possible to justify the mechanisms underlying the cause–effect relationship between an independent and a dependent variable [66]. Considering the previously hypothesized relationships and mediation models from the literature [53], we make the following mediation hypotheses:

**Hypothesis** **8** **(H8).** *Resource volume mediates the relationship between expenditure and patient satisfaction.*

**Hypothesis** **9** **(H9).** *Mortality level mediates the relationship between resource volume and patient satisfaction.*

Patient characteristics (age, gender, and social and economic status) affect the perception of health service provider quality, and therefore satisfaction [21,55,67]. The complexity of measuring patient satisfaction, mentioned above, is amplified by demographic heterogeneity [36].

The elevated role of doctors in the health system is indisputable. According to another study, we can observe higher satisfaction with family doctors than specialist doctors [68]. This may be due to more personal and closer relationships with family doctors than specialists [59]. In the doctor’s primary health, confidence, and security significantly influence patient satisfaction, and women are the most satisfied [12,67]. That distinction by gender is not significant with specialist doctors, indicated by a study of non-clinical factors [3]. Chang relates satisfaction with three elements: structure, process, and outcomes. In terms of process, it is observed that women are more satisfied than men, while, with the other two points, the difference between the sexes is not significant [69]. Valls and Parra [70] studied patient satisfaction with primary care doctors distinguishing by gender and found differences between men and women. Social role theory suggests that women are different from men in their nurturing (education) rather than their nature [71], and this could lead to an unequal perception of healthcare services. Based on previous studies and arguments, we state the last hypothesis of this empirical research:

**Hypothesis** **10** **(H10).** *Satisfaction of men and women is not configured in the same way.*

## 2. Materials and Methodology 

### 2.1. Sample and Data Collection

The Spanish Ministry of Health, Consumption and Social Welfare publishes on its website the so-called Key Indicators of the National Health System, known as INCLASS. These key indicators are an attempt to provide a picture of the health status of the population (mortality), the determinants of health (behavioral factors and living conditions), the response of the health system to the population’s needs (indicators that depend on the system: resources, level of use, expenditure and quality, as measured by patient satisfaction with healthcare received), and sociodemographic information (economic level). The conceptual model on which they are based is the one exemplified by the European Core Health Indicators (ECHI), formerly known as the European Community Health Indicators, which resulted from long-term cooperation between EU countries and the European Commission.

Therefore, we used secondary data, since they were obtained from the ministry’s official database. Information on expenditure, resources, level of use, and mortality is known to the public administration that manages the NHS. GDP data were obtained from the Spanish National Institute of Statistics. Finally, data on patient satisfaction provided by the Spanish Ministry of Health came from a survey called the Health Barometer, carried out by the National Institute of Statistics [72]. Three satisfaction variables were measured using a Likert scale ranging from 1 [very dissatisfied) to 10 (totally satisfied). According to officially published information, the data were obtained through direct surveys of citizens, but we do not know the specific procedure or the number of respondents. We worked with the information contained in the database, which corresponds to average values by autonomous community, differentiating by sex.

Spain comprises 17 autonomous communities plus the autonomous cities of Ceuta and Melilla, and the data reflect the annual average of indicators for each territorial unit. The study considered data from the period 2005–2018, except 2014, since there were no data for one of the variables: degree of satisfaction with the knowledge of the history and monitoring of health problems by family doctors and pediatricians. Moreover, we excluded from the study the autonomous cities, since there were no data on expenditure variables, which were fundamental to the study. Therefore, the final sample comprised 221 observations (17 autonomous communities over 13 years) for each study, i.e., 221 observations for men and 221 observations for women.

According to the statistical program G*Power (v. 3.1.9.6, Kiel, Germany), we calculated the necessary size of the sample [73] by considering a significance level of 0.05 and an effect size *f*^2^ of 0.15. We needed a sample of 114 observations for a statistical power of 0.8, which is the minimum power demanded in social and behavioral research. Even for statistical power of 0.95, the required sample of 166 observations is less than the 221 used here. Therefore, our sample was appropriate.

### 2.2. Measurement Variables

We considered all variables as composites and a set of indicators to integrate each composite or construct as a dimension of it [74]. Constructs are usually not one-dimensional but require several indicators to represent different facets [75]. Thus, removing an indicator from the measurement model alters the meaning of the construct [76]. In principle, the model does not impose any assumptions on the correlations between the indicators. Table 1 summarizes the composites and their indicators.

The final construct (dependent variable), patient satisfaction, was estimated in mode A, since indicators should be highly correlated, based on the idea that the construct causes covariation of the indicators [66]. We considered 3 indicators of patient satisfaction, measured on a Likert scale, ranging from 1 (least satisfied) to 10 (most satisfied): first, the degree of satisfaction with the functioning of the public health system, in general (PS1); second, the degree of satisfaction with the knowledge of the history and monitoring of health problems by family doctors and pediatricians (PS2); and third, the degree of satisfaction with the information received at specialists offices about health problems (PS3).

It should be noted that the quality of healthcare services is usually identified by patient satisfaction, and patients demand more information about their diagnosis and participate in deciding on the most appropriate treatment. Moreover, the results of a specific management policy can be measured through the evolution of patient satisfaction. Hence, its importance in resource management.

The representative constructs of expenses, resources, extent of use, and mortality were estimated in mode B, in which case the indicators were not expected to be strongly correlated. This mode indicates a causal relationship between the indicators and the construct.

Expenses are different across the country, since, in Spain, health management competencies are transferred to the autonomous communities; that is, they do not belong to the central government. Therefore, the level of expenditure and distribution of funds are not the same throughout the country. It is necessary to consider that the amount of expenditure influences the possibility to provide quality service. Public budgets are limited, especially in times of crisis like the present, while resource needs are increasing with growing technology, an aging population, and the diseases that come with economic development, with the stresses of daily life and environmental pollution. The 7 indicators of expenses include public health expenditure managed by the autonomous communities per protected inhabitant (EX1) and the percentage of this amount corresponding to the different expenditure items: specialty care services (EX2), primary care (EX3), concerts (outsourced expenses) (EX4), intermediate consumption (EX5), staff remuneration for the training of residents (EX6), and pharmacy spending (EX7).

Resources in each autonomous community depend not only on current spending but also on past spending. In other words, the policies applied in the past influence the possibilities of the present. There are nine indicators that make up the resource composite: for every 1000 inhabitants, medical personnel in specialized care (RE1), primary care medical staff (RE2), skilled care nurses (RE3), primary care nurses (RE4), running hospital beds (RE5), and day hospital posts (RE5) and for every 100,000 inhabitants, operating theaters (RE7), operating computed tomography (CT) (RE8), and nuclear magnetic resonance (NMR) (RE9).

In turn, the volume of available resources can determine the level of use of these resources by citizens. The study considered 7 indicators representative of the extent of use: for every 1000 inhabitants, the frequency of specialized attention consultations (EU1), frequency of hospital admissions (EU2), surgical intervention rate (EU4), CT usage rate (EU6), and NMR usage rate (EU7), as well as the number of days of an average hospital stay (EU3) and the outpatient surgery percentage (EU5).

It is logical to think, a priori, that the level of available health resources will influence mortality, and will also be a determining factor in patient satisfaction. Given the impossibility of contemplating all the possible causes of death, the construct was built with 4 of the most important ones dependent on the NHS: for every 100,000 inhabitants, the age-adjusted mortality rate for Alzheimer’s disease (MO1), cancer (MO2), diabetes mellitus (MO3), and cerebrovascular disease (MO4).

Finally, we used a control variable, the economic driver, measured as gross domestic product (GDP) per capita, considering its influence on patient satisfaction.

**Figure 1:**
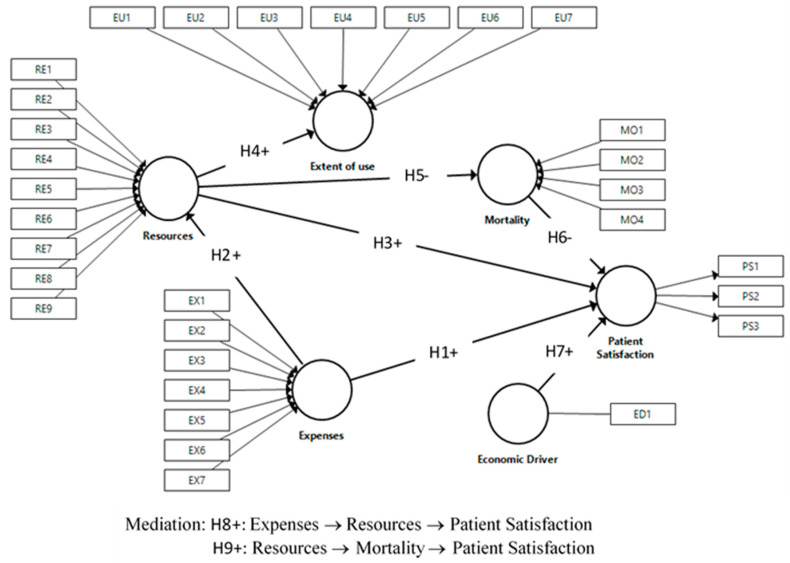
Research model and hypotheses. PS: Patient Satisfaction, EX: Expenses, RE: Resources, EU: Extent of use; MO: Mortality, ED: Economic driver.

The conceptual model represented in Figure 1 shows the relationships between the variables considered, reflecting the hypotheses given above.

### 2.3. Data Analysis

The technique chosen to assess the proposed research model was partial least squares structural equation modelling (PLS-SEM), which can test the relationship between the structural model constructs and the measurement model indicators. The statistical program used to perform the study was SmartPLS (v. 3.3.2.) [77], which also allowed implementing multi-group analysis (MGA) and the required measurement invariance of composite models (MICOM) to test the possible differences between men and women. MGA applies nonparametric SEM techniques [76,78,79,80]. PLS does not presuppose that the data should have a normal distribution. Instead, it uses a nonparametric bootstrap procedure to test the significance of the model’s coefficients [81] by extracting a high number of samples to replace the original sample. We created 5000 samples in this study [82].

First, the measurement model for the reflective construct (mode A) is assessed by analyzing the reliability of each indicator and the reliability, convergent validity, and discriminant validity of the construct. In the case of formative constructs (mode B), the multi-collinearity among indicators and the relevance and significance of the weight of each indicator were analyzed.

Second, the structural model was evaluated by analyzing the collinearity of the previous constructions, the sign, magnitude, and significance of the path coefficients, the coefficient of determination, the size of the effects, and, applying the blindfolding procedure, the predictive relevance of the model within the sample [83].

## 3. Results

According to the steps described in the above section, this section presents the developed study results. First, Table 2 shows the descriptive statistics of the indicators for the two considered samples, men and women. We can observe that expenses and resources are the same in both samples since there is no difference by gender. However, the extent of use is different in practice for men and women, but, in this study, the data discriminate only in the case of average hospital stay (higher in men) and outpatient surgery percentage (higher in women). However, mortality and patient satisfaction are different by gender.

Concerning mortality, average mortality from cancer, diabetes, and cerebrovascular disease is higher in men, while average mortality from Alzheimer’s is higher in women. The most remarkable difference by gender is cancer, for which average mortality is more than double in men than in women. In terms of satisfaction indicators, the differences by gender are small. However, on average, women are more satisfied with family doctors and men with specialist doctors and the NHS as a whole.

### 3.1. Measurement Model

#### 3.1.1. Composite Mode A

The composite measurement model in mode A (patient satisfaction) requires validation of individual item reliability, construct reliability, convergent validity, and discriminant validity (see Table 3).

The individual reliability of items is examined through the simple load or correlation with its construct, which has to be greater than 0.707 [84]. Effectively, panel A shows that indicators PS1, PS2, and PS3 exceed the required value in both samples, men and women.

Construct reliability describes the rigor with which the indicators measure the same construct. It is measured by Cronbach’s alpha, Dijkstra–Henseler’s rho, and composite reliability, which must be greater than 0.7 but less than 0.95 [85,86]. Panel B shows the values for the two studies, all of which are within the correct range. Convergent validity describes the degree to which a construct converges in explaining the variation of its indicators [83], and is measured by the average variance extracted (AVE), which has to be greater than or equal to 0.5 [87]. The patient satisfaction construct explained 78% of the variance of the assigned indicators for men and 78.5% for women.

The Fornell–Larcker criterion and the Heterotrait–Monotrait ratio (HTMT) allow us to verify the discriminant validity, which describes to what extent the patient satisfaction construct is empirically different from the other constructs of the structural model. According to the Fornell–Larcker criterion, for the reflective construct, the square root of the AVE (in bold) must be greater than the correlations between patient satisfaction and other constructs (in the horizontal and vertical lines) [78]. Both analyses met this requirement, as shown in panel C. Finally, the HTMT ratio exceeds the Fornell–Larcker criterion to detect the lack of discriminant validity [76]. This ratio has to be lower than 0.85, and neither 0.9 nor 1 should be in the confidence interval [2.5–97.5]. Panel D shows the correction of the patient satisfaction construct in the proposed model.

#### 3.1.2. Composite Mode B

The composite measurement model in mode B requires us to analyze the existence of possible collinearity between indicators, as well as the significance and relevance of outer weights.

In the context of PLS-SEM, there are problems of collinearity when the variance inflation factor (VIF) is equal to or greater than 5 [82], although some authors suggest a maximum value of 3.3 [88]. In this study, the value was always under the maximum of 5 for both men and women, as can be seen in Table 4 (estimated constructs in mode B for men) and Table 5 (estimated constructs in mode B for women). Therefore, we can say that there are no severe problems of collinearity.

The weights provide information on the contribution of each indicator to its respective construct. When the weight is not significant if the loading was significant, the indicator should be kept in the formative measurement model. Therefore, all indicators in the sample of men met the requirement to remain in the model. On the contrary, if the loading is low (less than 0.1) and not significant, the indicator should be removed [89]. In the case of women, indicator EX4 was just within the limit. However, we decided to keep it for comparative purposes to perform the MGA later.

The value and sign of the weights inform us about the contribution of indicators to the construct. Indicators with higher weights have more influence on the construct, and therefore on patient satisfaction. For example, public health expenditure per protected inhabitant (EX1) was the most influential on the expenses construct (0.579). Also remarkable is the negative sign of the indicator representing pharmacy spending (−0.323). We can interpret the rest of the indicators similarly.

### 3.2. Structural Model

**Figure 2:**
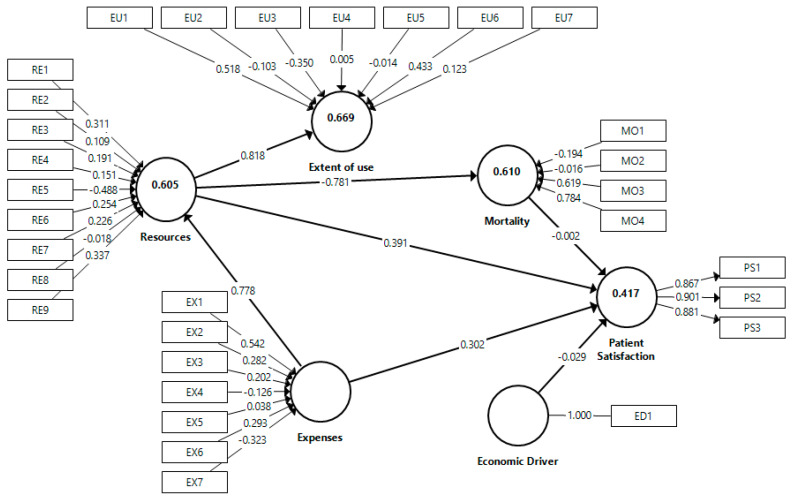
Whole model results for men.

**Figure 3:**
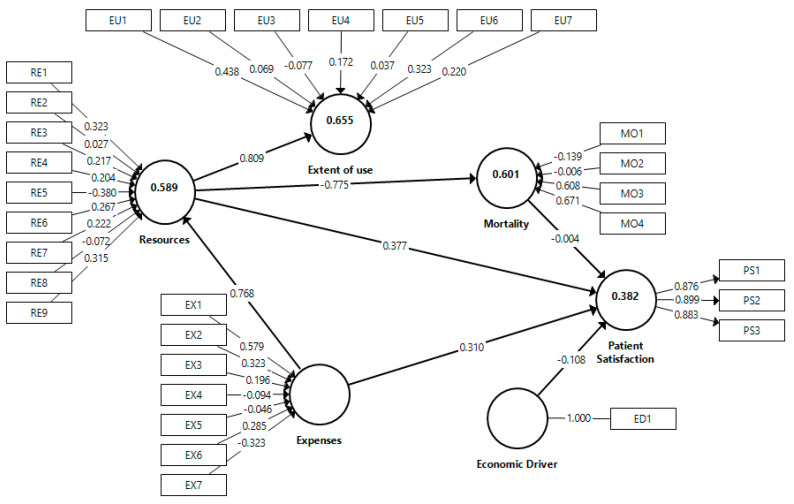
Whole model results for women.

In the second step, we assessed the structural model for the two groups. Table 6 and Figure 2 shows the results for men, and Table 7 and Figure 3 for women, which are similar.

Once it was proven that there were no collinearity problems, we analyzed the sign, magnitude (between +1 and –1, since these are standardized values), and statistical significance of the path coefficients. A two-tailed test was used in the bootstrapping to determine significance [90].

The results indicated that the economic driver (GDP), our control variable, had no significant effects on patient satisfaction for men (*p* = 0.580), but a negative effect, with a 90% significance level, for women (*p* = 0.080). Therefore, H7 was not supported in the sample of men and was weakly supported in the sample of women.

Expenses and resources had a positive and significant effect on patient satisfaction in both samples (*p* = 0.001 and 0.000, respectively, for men, and *p* = 0.005 and 0.000, respectively, for women). Thus, H1 and H3 were supported. Moreover, expenses also showed a positive and significant effect on resources (*p* = 0.000 for men and women), supporting H2. Therefore, H8 is supported since both the direct and indirect effects of expenses on patient satisfaction are significant and have the same sign, causing complementary mediation [91].

Resources had a negative and significant effect on mortality (*p* = 0.000 for men and women), supporting H5. On the contrary, mortality showed no significant effect on patient satisfaction (*p* = 0.987 for men and 0.975 for women), so H6 was not confirmed, and, consequently, the mediation effect represented by H9 was also not supported.

Furthermore, resources showed a positive and significant effect on the extent of use (*p* = 0.000 for men and women), confirming H4.

The coefficient of determination *R*^2^ is a measure of the explanatory capacity of the model. It represents the amount of variance of a construct explained by previous predictive constructs. Its value ranges between 0 and 1, so the higher it is, the more predictive capacity the model has for that construct. The results of the proposed model in the two samples are moderated [66], a little higher in the case of men. Specifically, in the sample of men, the value of *R*^2^ is 0.417, 0.605, 0.669, and 0.610 for patient satisfaction, resources, extent of use, and mortality, respectively; in the sample of women, the values are 0.382, 0.589, 0.655, and 0.601, respectively.

Effect size, determined by *f*^2^, is the degree to which an exogenous construct helps to explain a given endogenous construct in terms of *R*^2^. If *f*^2^ is less than or equal to 0.02, there is no effect [66], which happens for the economic driver (GDP) and mortality over patient satisfaction; hence, the path is not significant. When *f*^2^ is between 0.02 and 0.15, the effect is small, resulting in expenses and resources over patient satisfaction (0.058 and 0.074, respectively for men, and 0.059 and 0.068, respectively, for women). There are no moderate effects in the study because there are no *f*^2^ values between 0.15 and 0.35. However, there are three large effects in which *f*^2^ exceeds 0.35: expenses on resources (1.535 for men and 1.434 for women), resources on the extent of use (2.022 for men and 1.894 for women), and resources on mortality (1.566 for men and 1.503 for women). Therefore, the results are similar in both samples.

The Stone–Geisser test (*Q*^2^) measures the predictive relevance of reflective dependent constructs, in this study, the construct representing patient satisfaction. It is not a measure of prediction outside the sample, but indicates the extent to which the proposed model can predict the original observed values [92]. It uses a procedure called blindfolding, which consists of estimating the parameters by omitting part of the data of a given construct and then estimating the omitted data using the mean and the parameters of the previously estimated model [83]. *Q*^2^ values between 0.25 and 0.5, as in the case of the analyzed samples (0.306 for men and 0.282 for women), indicate average predictive relevance.

### 3.3. Multi-Group Analysis (MGA)

To analyze the significant differences between men and women in the proposed model, we performed MGA. This procedure requires prior application of measurement invariance of composite models (MICOM), by using a permutation test [76,93].

MICOM involves a three-step process: configuration invariance, compositional invariance, and equality of mean and variance of composites. Configuration invariance ensures that the compounds are specified equally in both groups. Since we used the same indicators in the two measurement models, we treated the data equally, and the algorithm was equally configured. Table 8 shows the results of the two remaining steps. Compositional invariance was achieved, since the original correlation was greater than or equal to 5% and all *p*-values are higher than 0.05 (*p*-values have not been reported for simplicity), assuring that the composites were formed in the same way in the two groups analyzed. Finally, the equality of mean and variance of composites was verified, since all differences were within the confidence interval (equally, all *p*-values were higher than 0.05); therefore, there is complete measurement invariance, and it is possible to apply MGA.

Table 9 shows the results of Henseler’s multi-group analysis [80] to assess if the differences between path coefficients in the samples of men and women are significantly different. This procedure is based on bootstrapping, and, when the *p*-value is lower than 0.05 or higher than 0.95, the path coefficients are different at the 5% significance level. H10 states that satisfaction is configured differently by men and women, but it does not establish what form the difference takes. Therefore, a two-tailed test was applied. The direct and indirect effects of expenses on patient satisfaction are significantly higher for women than men (*p*-value = 0.951 for the direct effect and 0.992 for the indirect effect), which indicates that women value spending more when judging health system quality. In terms of available resources, their indirect effect, through mortality, on health system quality is significantly higher for women than for men (*p*-value = 0.991). Finally, women also value more, in a significant way, the influence of mortality on patient satisfaction (*p*-value = 0.991). In this case, the difference is positive, but, as the paths are negative, the lower the mortality rate, the more the health system quality is valued by women than men.

## 4. Discussion

After analyzing 31 variables to evaluate their influence on patient satisfaction in Spain, we found relevant information. Data were obtained from the Spanish Ministry of Health, Consumption and Social Welfare for the entire Spanish territory between 2005 and 2018, except 2014. The applied technique was partial least squares structural equation modeling (PLS-SEM). A positive relationship between the constructs of expenditure and volume of resources and patient satisfaction was confirmed, as well as the influence of resource allocation on the extent of use. However, the levels of mortality analyzed (Alzheimer’s, cancer, diabetes, and cerebrovascular disease) did not influence the perception of healthcare system quality. GDP was also not relevant. Regarding indicators, public health expenditure, spending on primary and specialist care services, expenses for training of resident doctors, the number of NMR machines, day hospital posts, operating theaters, skilled and primary care nurses, and specialized and primary care doctors, and Alzheimer’s mortality rate had a positive and significant influence on patient satisfaction in both study groups. In contrast, pharmacy spending, subcontracts with private healthcare system (concerts), running hospital beds, diabetes, and cerebrovascular mortality negatively influenced patient satisfaction in both groups.

The empirical analysis can provide healthcare managers with adequate information for decision-making and help to improve health system quality, which is decisive in the current context, characterized by extreme competition, globalization, and increasing demand.

Most of the previous patient satisfaction research was aimed at improving the practice of medical and nursing staff [3,6,17,42]. Thus, this study’s variables refer mainly to the direction of investment and budgeting of expenses. Research on resource management and strategic direction is scarce, and even more so when referring to studies distinguished by gender [59,94]. In our investigation, we found that, overall, women are more sensitive than men to the volume of expenditures and resources invested by the public administration. In other words, women are more sensitive to improvements in the quality of the healthcare system resulting from greater financial resources.

In line with previous literature, we found that men and women value family and specialist doctors more than the health system as a whole [53,68,69]. In contrast, women are more satisfied with family doctors and men with specialist doctors. Although Morales’s study indicated that greater satisfaction was observed with family doctors than specialist doctors [68], we can now affirm that it is not always this way; it depends on the sex of the patient.

This study analyzed the influence of volume resources, expenditures, and mortality on patient satisfaction. Moreover, it analyzed the relationship between resource allocation and the degree of use of the Spanish health system. The control variable introduced in the model was the economic driver, represented by GPD per capita. The model explained the latent variable patient satisfaction by 41.7% for the sample of men and 38.2% for the sample of women. Expenses and resource constructs had a positive and significant influence on patient satisfaction, while mortality had no significant effect.

Regarding resource allocation, previous studies emphasized that patient satisfaction increases with investment in technological equipment, infrastructure [9,25,40], and qualified doctors [29]. In line with those findings, we show that citizens perceive the quality of the healthcare system as higher when the number of healthcare personnel (doctors and nurses) increases, and place higher value on those who provide specialized care than those who provide primary care. Likewise, a greater number of operating rooms and NMR machines increase satisfaction. Paradoxically, the same does not happen with CT machines. That may be because the correct reallocation of resources does not harm service quality, and therefore does not harm satisfaction. However, a positive sign was observed for the number of day hospital posts and a negative sign for the number of hospital beds, in line with the study by Xesfingi and Vozikis [95]. This indicates that people prefer (when the severity of the disease or surgery allows it) to be cared for in an outpatient rather than in inpatient setting. A previous study indicated that there was a positive relationship between ambulatory surgery and patient satisfaction [59]. Men’s and women’s behavior is similar.

For the expenditures’ construct, the indicator with the most positive weight was public health expenditure per inhabitant. With regard to the distribution of expenditures, patients value above all spending on specialized care, including on resident physicians, i.e., specialty physicians in training. Spending on primary care is also positively valued. Conversely, pharmacy spending showed a negative relationship with patient satisfaction in both study groups. That is contrary to Fenton’s study, where drug prescriptions and satisfaction had a positive influence [60]. However, it is in line with previous studies [53,59]. In this respect, Pascoe [96] found that medication expenditure was satisfactory only for patients over 65 years of age. This means that the younger population prefers other types of treatment or more natural therapies instead of traditional medication. The Spanish heath system has the authority to resort to the private sector when there are insufficient public resources to meet the demand. This outsourcing process is known as a concert. Regarding the expenditure dedicated to concerts, the influence is negative and only significant for men. This indicates that the population prefers to be treated in public rather than private hospitals.

Regarding mortality and its cases, the results showed that cancer mortality did not have a significant influence on satisfaction. However, Alzheimer’s mortality is valued positively. This may be because caregivers of people with advanced dementia, who are unable to communicate or move, are relieved when they die. Another interpretation could be that since it is a disease without a cure, it does not depend on the quality of the health system’s services, or it is a death associated with old age.

On the other hand, diabetes and cardiovascular mortality negatively influence patient satisfaction. This means that citizens understand that an advanced healthcare system should provide the necessary care to control the progression of these diseases and avoid a fatal outcome. A previous study indicated that women reported a significantly higher impact of diabetes on quality of life and more restlessness regarding this issue than men [97]. However, we did not find significant differences by gender regarding the influence of diabetes mortality on patient satisfaction. That could be due to the significant and positive impact of the degree of disease control in determining health-related quality of life [97]. It is also essential that people with diabetes receive integral care to prevent other diseases associated with it [98]. However, our research concluded that variation in the mortality construct had a more significant influence on satisfaction when the group analyzed was women. Historically, cardiovascular disease has been associated with older adults, but this pattern has changed in recent times, and it is increasingly common for young adults to die from this disease. A more modern health system, emphasizing prevention and detection, treatment, and control, would be valued by the population [99].

The contribution of resource allocation to determining the extent of use was related, first, with frequency of specialist consultation and then CT equipment usage, for both men and women. An economic recession has direct consequences to health, increasing restricted budgets, lengthening waiting times for treatment (due to lack of equipment), and increasing medical consultations with specialists (due to lack of personnel). Furthermore, the relationship between resource allocation and mortality is significant and positive. Previous studies indicated that it is possible to reduce spending, increase income, and, at the same time, improve mortality rates [100]

As mentioned above, the control variable was represented by GDP per capita, which had a negative effect on satisfaction, but not significant, for men and was at a less than 10% significance level for women. The negative relationship implies that people with higher income are more demanding with the healthcare system. The previous literature is mixed, since some studies found a significant and positive relationship [65,67], others a negative relationship [1], and still others no relationship [53].

Per capita health expenditure in Spain is below the European Union average, even though social inequalities are less pronounced than in many countries on the continent [101]. The Spanish health system is not homogeneous throughout its territory, since, as mentioned above, healthcare competencies are transferred to the autonomous communities, which is reflected in the efficiency of public health services. The ultimate goal of a health system is to improve citizens’ health and quality of life, but political, social, cultural, and economic issues inevitably have an influence. Conducting a proper management analysis of intrinsic and perceived quality helps managers and institutions to meet their objectives [100].

A quality healthcare system will require prioritizing investment in primary care and, above all, specialized care. It will need to invest in hiring a large number of doctors and nurses, as well as doctors in training. It will also need to have high-level equipment, such as NMR machines. Day hospital positions should be prioritized over the number of hospital beds. It is necessary to expand the capacity to care for patients in the public system and not refer them to private hospitals. Drug prescriptions should be reduced, and patients should be given the option to use alternative therapies, especially younger patients.

Numerous international studies deal with the subject of patient satisfaction, although most of them examine indicators of behavior and suitability of the doctor. Studies on resource management are scarce in Spain, mainly due to the lack of data [63,74]. In this empirical study, the patient satisfaction construct explained 78.5% of the variance in the case of women and 78% in men. The rest of the variance could be explained by variables not considered in the model, such as patient participation in the diagnostic process [49], the regularity with which patients are monitored [11], and physicians behaving with courtesy and respect [23], among others. We consider it convenient to expand the research carried out by influencing variables such as life expectancy at birth and infant mortality. It would also be useful to study the influence of educational level, geographic region, and poverty rate. Therefore, the main limitation of the study is the availability of data. Including additional variables, such as those mentioned above, as well as having all patient responses, not just the averages for each autonomous community per year, would undoubtedly allow us to obtain stronger results and conclusions. Many satisfaction studies are conducted in specific hospitals. However, we are convinced that studies such as this one, carried out at the national level, are necessary. For this, researchers need transparency in public information, i.e., publicly available data.

## 5. Conclusions

The evident growing need for accurate and integral information to fulfill organizational objectives (support strategic planning and control) makes the usefulness of this research unquestionable. As we were able to confirm, any decision having to do with resource allocation and expenditure within the health system directly affects patient satisfaction. An ex-post analysis was carried out using reliable data extracted from the Spanish Ministry of Health, Consumption, and Social Welfare using the structural equation modelling approach.

This study shows that the level of expenditure has a direct influence on patient satisfaction, and therefore on the quality of the healthcare system. It is important to increase spending on primary care, but especially on specialized medical care and diagnostic equipment. In addition, reducing the use of drugs in favor of alternative treatments or therapies is considered to be positive. Likewise, spending has an impact on available resources and these, in turn, have a positive influence on the level of use and a negative impact on mortality. Resources, especially healthcare staff, NRM equipment, and the number of posts in day hospitals, increase patients’ perception of the NHS.

Regarding gender, and apart from differences in specific variables, in general terms, women are more sensitive to public investment in health than men.

## Figures and Tables

**Table 1:** Composites and descriptions of indicators.

Composite	Indicators	Description
Patient satisfaction (mode A)	PS1	Degree of satisfaction with functioning of public health system
	PS2	Degree of satisfaction with knowledge of history and monitoring of health problems by family doctors and pediatricians
	PS3	Degree of satisfaction with information received at specialists’ offices about health problems
Expenses (mode B)	EX1	Public health expenditure managed by autonomous community per protected inhabitant
	EX2	Percentage of spending on specialized care services
	EX3	Percentage of spending on primary care
	EX4	Percentage of spending dedicated to concerts
	EX5	Percentage of spending on intermediate consumption
	EX6	Percentage of public health expenditure on staff remuneration for training of residents
	EX7	Percentage of pharmacy spending
Resources (mode B)	RE1	Medical personnel in specialized care per 1000 inhabitants
	RE2	Primary care medical staff per 1000 people assigned
	RE3	Skilled care nurses per 1000 inhabitants
	RE4	Primary care nurses per 1000 people assigned
	RE5	Running hospital beds per 1000 inhabitants
	RE6	Day hospital posts per 1000 inhabitants
	RE7	Operating theaters per 100,000 inhabitants
	RE8	Operating computed tomography (CT) equipment per 100,000 inhabitants
	RE9	Nuclear magnetic resonance (NMR) per 100,000 inhabitants
Extent of use (mode B)	EU1	Frequency of specialized attention consultations per 1000 inhabitants/year
	EU2	Frequency of hospital admissions per 1000 inhabitants/year
	EU3	Number of days of average hospital stay
	EU4	Surgical intervention rate per 1000 inhabitants/year
	EU5	Outpatient surgery percentage
	EU6	CT usage rate per 1000 inhabitants/year
	EU7	NMR usage rate per 1000 inhabitants/year
Mortality (mode B)	MO1	Age-adjusted mortality rate for Alzheimer’s disease per 100,000 inhabitants
	MO2	Age-adjusted death rate from cancer per 100,000 inhabitants
	MO3	Age-adjusted mortality rate for diabetes mellitus per 100,000 inhabitants
	MO4	Age-adjusted death rate from cerebrovascular disease per 100,000 inhabitants
Economic driver	ED1	Gross domestic product (GDP) per capita

PS: Patient Satisfaction, EX: Expenses, RE: Resources, EU: Extent of use; MO: Mortality, ED: Economic driver.

**Table 2:** Descriptive statistics.

Construct and Associated Indicators	Men		Women	
	Mean	Standard Deviation	Mean	Standard Deviation
Patient satisfaction (PS)				
PS1	6.572	0.439	6.533	0.461
PS2	7.365	0.424	7.481	0.402
PS3	7.293	0.425	7.275	0.442
Expenses (EX)				
EX1	1415.785	167.139	1415.785	167.139
EX2	58.902	4.901	58.902	4.901
EX3	13.969	1.748	13.969	1.748
EX4	7.314	5.210	7.314	5.210
EX5	22.867	4.437	22.867	4.437
EX6	3.235	0.908	3.235	0.908
EX7	18.546	3.034	18.546	3.034
Resources (RE)				
RE1	1.698	0.221	1.698	0.221
RE2	0.778	0.106	0.778	0.106
RE3	2.931	0.446	2.931	0.446
RE4	0.661	0.108	0.661	0.108
RE5	2.497	0.457	2.497	0.457
RE6	0.274	0.128	0.274	0.128
RE7	6.438	1.016	6.438	1.016
RE8	1.136	0.260	1.136	0.260
RE9	0.558	0.224	0.558	0.224
Extent of use (EU)				
EU1	1619.646	244.892	1619.646	244.892
EU2	91.937	15.430	91.937	15.430
EU3	7.897	0.976	6.600	0.703
EU4	69.709	14.584	69.709	14.584
EU5	39.609	8.462	41.537	8.251
EU6	72.408	17.468	72.408	17.468
EU7	28.395	14.788	28.395	14.788
Mortality (MO)				
MO1	9.477	2.407	12.645	3.127
MO2	213.869	20.038	100.495	6.233
MO3	12.611	6.219	10.081	5.540
MO4	36.937	9.981	28.790	8.318
Economic driver (ED)				
ED1	22.987	4.578	22.987	4.578

**Table 3:** Assessment of measurement model. Estimated constructs in mode A.

**(A) Outer Loadings**								
**Indicator**	**Men**				**Women**			
PS1	0.881				0.883			
PS2	0.901				0.899			
PS3	0.867				0.876			
**(B) Construct Reliability and Average Variance Extracted**								
**Composite**	**Cronbach’s Alpha**		**Dijkstra–Henseler’s Rho**		**Composite Reliability (CR)**		**AVE**	
Variable	Men	Women	Men	Women	Men	Women	Men	Women
Patient satisfaction	0.860	0.864	0.874	0.875	0.914	0.916	0.780	0.785
**(C) Discriminant Validity (Fornell–Larcker Criterion)**								
**Group**	**Variable**		**DE**	**EX**	**MO**	**RE**	**PS**	**EU**
Men	DE		1.000					
	EX		0.305	n.a.				
	MO		−0.459	−0.700	n.a.			
	RE		0.309	0.778	−0.781	n.a.		
	PS		0.185	0.598	−0.505	0.618	**0.883**	
	EU		0.311	0.580	−0.692	0.818	0.447	
Women	DE		1					
	EX		0.339	n.a.				
	MO		−0.551	−0.703	n.a.			
	RE		0.351	0.768	−0.775	n.a.		
	PS		0.131	0.566	−0.454	0.580	**0.886**	
	EU		0.337	0.535	−0.656	0.809	0.395	
**(D) Discriminant Validity (HTMT Criterion)**								
**PS** **→** **DE**	**Original Sample**		**Sample Mean**		**CI Lo2.5%**		**CI Hi97.5%**	
Men	0.193		0.208		0.129		0.275	
Women	0.140		0.162		0.083		0.182	

AVE: Average Variance Extracted. PS: Patient Satisfaction. DE: Economic Driver. HTMT: Heterotrait-Monotrait. CI Lo and CI Hi: Confidence Interval, Low and High, respectively. In the mode A composite, the amount of variance that a construct captures from its indicators must be greater than the variance that the construct shares with other constructs.

**Table 4:** Assessment of measurement model. Estimated constructs in mode B for men.

Variables	VIF	Weights	*t*	CI 2.5%	CI 97.5%	Loadings
Expenses						
EX1	1.385	0.542 ***	7.265	0.395	0.686	0.731 ***
EX2	3.559	0.282 ***	2.797	0.094	0.491	0.604 ***
EX3	1.292	0.202 ***	3.073	0.068	0.327	0.073 ^‡^
EX4	1.363	−0.126 **	2.292	−0.233	−0.018	−0.156 **
EX5	2.536	0.038 ^‡^	0.371	−0.189	0.223	0.552 ***
EX6	1.610	0.293 ***	3.867	0.157	0.454	0.456 ***
EX7	2.316	−0.323 ***	3.637	−0.494	−0.143	−0.754 ***
Resources						
RE1	4.345	0.311 ***	3.556	0.155	0.502	0.799 ***
RE2	3.704	0.109 ^‡^	1.426	−0.036	0.264	0.304 ***
RE3	3.604	0.191 **	2.451	0.044	0.354	0.723 ***
RE4	4.674	0.151 *	1.646	−0.028	0.331	0.358 ***
RE5	1.797	−0.488 ***	6.725	−0.626	−0.341	0.162 *
RE6	1.831	0.254 ***	5.309	0.171	0.362	0.703 ***
RE7	3.412	0.226 ***	2.828	0.074	0.386	0.733 ***
RE8	2.734	−0.018 ^‡^	0.278	−0.149	0.098	0.636 ***
RE9	2.210	0.337 ***	4.938	0.211	0.479	0.807 ***
Extent of use						
EU1	1.751	0.518 ***	6.545	0.364	0.665	0.808 ***
EU2	2.487	−0.103 ^‡^	0.837	−0.353	0.129	0.511 ***
EU3	2.521	−0.350 ***	3.566	−0.535	−0.152	−0.538 ***
EU4	3.226	0.005 ^‡^	0.049	−0.171	0.240	0.667***
EU5	1.619	−0.014 ^‡^	0.188	−0.163	0.132	0.323 ***
EU6	3.039	0.433 ***	4.880	0.270	0.619	0.809 ***
EU7	3.222	0.123 ^‡^	1.324	−0.056	0.313	0.782 ***
Mortality						
MO1	1.162	−0.194 **	2.457	−0.347	−0.038	−0.028
MO2	1.694	−0.016 ^‡^	0.152	−0.232	0.187	0.772 ***
MO3	1.018	0.619 ***	7.125	0.438	0.771	0.636 ***
MO4	1.648	0.784 ***	9.966	0.638	0.947	0.777 ***

* *p* < 0.10; ** *p* < 0.05; *** *p* < 0.01; ^‡^, not significant. Significance, *t*-statistic, and 95% bias-corrected confidence interval performed by bootstrapping procedure with 5000 replications. VIF, variance inflation factor.

**Table 5:** Assessment of measurement model. Estimated constructs in mode B for women.

Variables	VIF	Weights	*t*	CI 2.5%	CI 97.5%	Loadings
Expenses						
EX1	1.385	0.579 ***	7.947	0.435	0.721	0.755 ***
EX2	3.559	0.323 ***	3.010	0.118	0.542	0.601 ***
EX3	1.292	0.196 ***	2.838	0.057	0.329	0.075 ^‡^
EX4	1.363	−0.094 ^‡^	1.444	−0.223	0.030	−0.100
EX5	2.536	−0.046 ^‡^	0.391	−0.296	0.167	0.489 ***
EX6	1.610	0.285 ***	3.648	0.149	0.453	0.411 ***
EX7	2.316	−0.323 ***	3.526	−0.497	−0.143	−0.773 ***
Resources						
RE1	4.345	0.323 ***	4.063	0.185	0.503	0.839 ***
RE2	3.704	0.027 ^‡^	0.337	−0.137	0.179	0.299 ***
RE3	3.604	0.217 ***	2.687	0.070	0.378	0.767 ***
RE4	4.674	0.204*	1.947	0.009	0.415	0.385 ***
RE5	1.797	−0.380 ***	5.368	−0.516	−0.239	0.254 ***
RE6	1.831	0.267 ***	5.528	0.182	0.374	0.731 ***
RE7	3.412	0.222 ***	2.568	0.063	0.403	0.750 ***
RE8	2.734	−0.072 ^‡^	1.063	−0.220	0.052	0.633 ***
RE9	2.210	0.315 ***	4.152	0.171	0.466	0.816 ***
Extent of use						
EU1	1.743	0.438 ***	5.549	0.293	0.587	0.826 ***
EU2	2.288	0.068 ^‡^	0.617	−0.159	0.293	0.593 ***
EU3	1.785	−0.077 ^‡^	0.823	−0.251	0.112	−0.211 **
EU4	3.189	0.172 ^‡^	1.405	−0.053	0.418	0.724 ***
EU5	1.708	−0.037 ^‡^	0.488	−0.118	0.180	0.465 ***
EU6	3.021	0.323 ***	3.489	0.146	0.510	0.818 ***
EU7	3.088	0.220 **	2.299	0.044	0.423	0.792 ***
Mortality						
MO1	1.082	−0.139 *	1.835	−0.282	0.013	−0.102
MO2	1.208	−0.006 ^‡^	0.076	−0.170	0.153	0.313 ***
MO3	1.161	0.608 ***	7.420	0.446	0.758	0.749 ***
MO4	1.058	0.671 ***	11.369	0.558	0.785	0.794 ***

* *p* < 0.10; ** *p* < 0.05; *** *p* < 0.01; ^‡^, not significant. Significance, *t* statistic, and 95% bias-corrected confidence interval performed by bootstrapping procedure with 5000 replications. VIF: variance inflation factor.

**Table 6:** Assessment of the structural model for men.

**(A) Direct Effects**							
**Effects**	**Path**	***t***	**CI 2.5%**	**CI 97.5%**		***f*** **^2^**	**VIF**
ED→PS	−0.029 ^‡^	0.560	−0.130	0.070		0.001	1.278
EX→PS	0.302 ***	3.109	0.101	0.488		0.058	2.684
RE→PS	0.391 ***	3.829	0.181	0.583		0.074	3.102
MO→PS	−0.002 ^‡^	0.016	−0.205	0.197		0	3.539
R^2^ = 0.417; Q^2^ = 0.306							
EX→RE	0.778 ***	31.679	0.714	0.816		1.535	1
R^2^ = 0.605; Q^2^ = 0.212							
RE→EU	0.818 ***	33.768	0.757	0.857		2.022	1
R^2^ = 0.669; Q^2^ = 0.289							
RE→MO	−0.781 ***	32.697	−0.821	−0.724		1.566	1
R^2^ = 0.610; Q^2^ = 0.203							
**(B) Specific Indirect Effects**							
**Effects**	**Path**	***t***	**CI 2.5%**		**CI 97.5%**		
EX→RE→MO	−0.608 ***	19.574	−0.658		−0.531		
RE→MO→PS	0.001 ^‡^	0.016	−0.155		0.163		
EX→RE→MO→PS	−0.001 ^‡^	0.016	−0.531		0.129		
EX→RE→PS	0.304 ***	3.811	0.137		0.451		
EX→RE→EU	0.636 ***	21.679	0.564		0.683		
**(C) Total Indirect Effects**							
EX→MO	−0.608 ***	19.574	−0.658		−0.531		
EX→PS	0.305 ***	3.842	0.132		0.448		
EX→EU	0.636 ***	21.679	0.564		0.683		
RE→PS	0.001 ^‡^	0.016	−0.155		0.163		

** *p* < 0.05; *** *p* < 0.01; ^‡^, not significant. Significance, *t* statistic, and 95% bias-corrected confidence interval performed by bootstrapping procedure with 5000 replications. VIF: variance inflation factor.

**Table 7:** Assessment of the structural model for women.

**(A) Direct Effects (Path Coefficients)**							
**Effects**	**Path**	***t***	**CI 2.5%**	**CI 97.5%**		***f*** **^2^**	**VIF**
ED→PS	−0.108 *	1.746	−0.227	0.016		0.013	1.467
EX→PS	0.310 ***	2.803	0.078	0.514		0.059	2.621
RE→PS	0.377 ***	3.512	0.157	0.579		0.068	3.367
MO→PS	−0.004 ^‡^	0.031	−0.243	0.234		0	3.521
R^2^ = 0.382; Q^2^ = 0.282							
EX→RE	0.768 ***	30.480	0.700	0.806		1.434	1
R^2^ = 0.589; Q^2^ = 0.215							
RE→EU	0.809 ***	33.118	0.745	0.846		1.894	1
R^2^ = 0.655; Q^2^ = 0.287							
RE→MO	−0.775 ***	32.131	−0.817	–0.721		1.503	1
R^2^ = 0.601; Q^2^ = 0.192							
**(B) Specific Indirect Effects**							
**Effects**	**Path**	**t**	**CI 2.5%**		**CI 97.5%**		
EX→RE→MO	−0.595 ***	19.476	−0.645		−0.522		
RE→MO→PS	0.003 ^‡^	0.031	−0.181		0.191		
EX→RE→MO→PS	−0.002 ^‡^	0.030	−0.142		0.149		
EX→RE→PS	0.289 ***	3.534	0.117		0.442		
EX→RE→EU	0.621 ***	21.257	0.548		0.666		
**(C) Total Indirect Effects**							
EX→MO	−0.595 ***	19.476	−0.645		−0.522		
EX→PS	0.291 ***	3.436	0.107		0.443		
EX→EU	0.621 ***	21.257	0.548		0.666		
RE→PS	0.003 ^‡^	0.031	−0.181		0.191		

* *p* < 0.10; *** *p* < 0.01; ^‡^, not significant. Significance, *t*-statistic, and 95% bias-corrected confidence interval performed by bootstrapping procedure with 5000 replications. VIF: variance inflation factor.

**Table 8:** Results of invariance measurement testing using permutation.

Construct	Configuration Invariance (Same Algorithms for Both Groups)	Compositional Invariance		Partial Measurement Invariance Established	Equal Mean Assessment				Equal Variance Assessment				Full Measurement Invariance Established
		Correlation Original	5%		Difference	CI 2.5%	CI 97.5%	Equal	Difference	CI 2.5%	CI 97.5%	Equal	
DE	Yes	1.000	1.000	Yes		−0.212	0.212			−0.226	0.219		
EX	Yes	0.996	0.952	Yes		−0.267	0.261			−0.281	0.282		
MO	Yes	0.998	0.941	Yes	0.087	−0.199	0.171	Yes	-0.013	−0.320	0.297	Yes	Yes
RE	Yes	0.993	0.972	Yes		−0.178	0.201			−0.254	0.238		
PS	Yes	1.000	0.999	Yes	−0.069	−0.180	0.191	Yes	-0.024	−0.246	0.256	Yes	Yes
EU	Yes	0.961	0.952	Yes	−0.127	−0.176	0.193	Yes	0.048	−0.239	0.243	Yes	Yes

CI: Confidence Interval.

**Table 9:** Henseler’s multi-group analysis (MGA).

Relationship	Men	Women	Difference	*p*-Value	Significant
**Panel A. Direct Effects (Path Coefficients)**					
ED→PS	−0.029	−0.108	0.079	0.319	No
EX→PS	0.302	0.310	−0.008 *	0.951	Yes
RE→PS	0.391	0.377	0.014	0.931	No
MO→PS	−0.002	−0.004	0.002 *	0.991	Yes
EX→RE	0.778	0.768	0.011	0.765	No
RE→EU	0.818	0.809	0.009	0.797	No
RE→MO	−0.781	−0.775	−0.006	0.850	No
**Panel B. Specific Indirect Effects**					
EX→RE→MO	−0.608	−0.595	−0.013	0.763	No
RE→MO→PS	0.001	0.003	−0.002 *	0.991	Yes
EX→RE→MO→PS	0.001	0.002	−0.001 *	0.992	Yes
EX→RE→PS	0.304	0.289	0.015	0.903	No
EX→RE→EU	0.636	0.621	0.016	0.711	No

Note: Difference is men vs. women. In Henseler’s MGA method, *p*-value lower than 0.05 or higher than 0.95 indicates significant differences at 5% level between specific path coefficients across two groups. * *p* < 0.05 or >0.95.

## Data Availability

Data publicly available from the Spanish Ministry of Health.
